# Comparability of Weighed Dietary Records and a Self-Administered Diet History Questionnaire for Estimating Monetary Cost of Dietary Energy

**DOI:** 10.4137/EHI.S1036

**Published:** 2008-09-15

**Authors:** Kentaro Murakami, Satoshi Sasaki, Yoshiko Takahashi, Hitomi Okubo, Naoko Hirota, Akiko Notsu, Mitsuru Fukui, Chigusa Date

**Affiliations:** 1 Department of Social and Preventive Epidemiology, School of Public Health, the University of Tokyo, Tokyo, Japan.; 2 Department of Health and Nutrition, School of Home Economics, Wayo Women’s University, Chiba, Japan.; 3 Department of Nutrition Sciences, Kagawa Nutrition University, Saitama, Japan.; 4 Department of Health and Nutritional Science, Faculty of Human Health Science, Matsumoto University, Nagano, Japan.; 5 Food Science and Nutrition Department, Tottori College, Tottori, Japan.; 6 Department of Statistics, Osaka City University Medical School, Osaka, Japan.; 7 Department of Food Science and Nutrition, Faculty of Human Life and Environment, Nara Women’s University, Nara, Japan.

**Keywords:** monetary diet cost, dietary record, diet history questionnaire, retail food price, epidemiology

## Abstract

An increasing number of studies have estimated monetary diet cost using various dietary assessment methods, based on databases on retail food prices, for investigating its association with dietary intake and health outcomes. However, information regarding the comparability of monetary diet cost across dietary assessment methods is absolutely lacking. This study compared monetary cost of dietary energy estimated from weighed dietary records (DRs) with that estimated from a self-administered diet history questionnaire (DHQ). The subjects were 92 Japanese women aged 31–69 years and 92 Japanese men aged 32–76 years. The DHQ (assessing diet during the preceding month) and 4-day DRs (one weekend day and three weekdays) were completed in each season over a 1-year period (DHQs1-4 and DRs1-4, respectively). An additional DHQ was completed at one year after completing DHQ1 (DHQ5). Monetary cost of dietary energy (Japanese yen/4184 kJ) was calculated using food intake information derived from each dietary assessment method, based on retail food prices. Pearson correlation between the mean of DRs1-4 and mean of DHQs1-4 was 0.64 for women and 0.69 for men. Pearson correlation between the mean of DRs1-4 and DHQ1 was 0.60 for women and 0.52 for men, while intraclass correlation between DHQ1 and DHQ5 was 0.64 for women and 0.51 for men. These data indicate reasonable comparability of monetary cost of dietary energy across DR and a DHQ as well as usefulness of a single administration of the DHQ for estimating monetary cost of dietary energy.

## Introduction

The price of food is undoubtedly an important determinant of food choice.^1^,^2^ An increasing number of studies have investigated the monetary cost of diet in relation to diet quality^3^–^17^ and health status variables such as body mass index.^14^,^15^ In these studies, monetary diet cost was consistently estimated using food intake information from either dietary assessment methods assessing foods actually consumed (such as 24-hour dietary recall^4^–^6^ and dietary records (DRs)^6^–^11^) or those retrospectively assessing dietary habits (such as diet history interview^12^,^13^ or questionnaire^14^ and food frequency questionnaires^15^–^17^), based on databases on retail food prices (with only one exception,^3^ where monetary diet cost was assessed based on estimated food expenditures from recall or actual food expenditure reports).

To our knowledge, however, the comparability of monetary diet cost across dietary assessment methods has not been assessed. Demonstration of basic information regarding the utility of monetary diet cost estimated based on food intake data will facilitate future research on the important public health topic of dietary cost, nutrient and food intake and health status. Here, we compared monetary cost of dietary energy estimated from weighed DRs with that estimated from a self-administered diet history questionnaire (DHQ).

## Methods

### Subjects

The present study was based on a survey conducted in three areas of Japan (i.e. Osaka (urban), Nagano (rural inland) and Tottori (rural coastal)). Detailed descriptions of the survey have been published elsewhere.^18^ Briefly, apparently healthy women aged 30–69 years who were willing to participate with their husbands were recruited in each area, such that each 10-year age class (30–39, 40–49, 50–59 and 60–69 years) contained eight women equally (without consideration of the age of the men), giving a total of 96 women and 96 men invitees. Group orientations for the subjects were held prior to the study, at which the study purpose and protocol were explained. Written informed consent was obtained from each subject. A total of 92 women aged 31–69 years and 92 men aged 32–76 years completed the study protocol and were included in the present analysis. Basic characteristics of the 92 women and 92 men have been described elsewhere.^18^

### Dietary assessment

Between November 2002 and September 2003, the subjects completed the DHQ (assessing diet during the preceding month) and the 4-nonconsecutive-day weighed DRs (one weekend day and three weekdays) four times (once per season) at intervals of approximately three months (DHQ1 in November 2002 (autumn), DHQ2 in February 2003 (winter), DHQ3 in May 2003 (spring) and DHQ4 in August and September 2003 (summer) and DR1 in November and December 2002 (autumn), DR2 in February 2003 (winter), DR3 in May 2003 (spring) and DR4 in August and September 2003 (summer)). In each season, the DHQ was completed before the start of the dietary recording period. An additional DHQ (DHQ5) was also completed about one year after completing DHQ1 (in November 2003 (autumn)).

Detailed descriptions of the DRs have been published elsewhere.^18^ Briefly, the subjects were asked to record and weigh all foods and drinks consumed on each recording day, and then to fax the completed records to the local staff (registered dietitians). The submitted forms were reviewed by the staff and, if necessary, the subjects were asked to add or modify the records by telephone or fax. The coding of records and conversion of other measurements of quantities into grams were performed by trained registered dietitians in the survey center in accordance with uniform procedures. A total of 1299 food and beverage items appeared in the DR. Estimates of daily energy intake were calculated based on the Standard Tables of Food Composition in Japan.^19^

Detailed descriptions of the DHQ have also been published elsewhere.^18^,^20^–^22^ Briefly, the DHQ is a 16-page structured questionnaire that assesses dietary habits during the preceding month (i.e. the consumption frequency and portion size of selected foods commonly consumed in Japan as well as general dietary behaviour and usual cooking methods).^20^ Responses to the DHQ were checked at least twice for completeness by the local staff, and when necessary reviewed with the subject to ensure the clarity of answers. Estimates of daily intake for foods (150 items in total) and energy were calculated using an ad hoc computer algorithm for the DHQ^18^,^20^ based on the Standard Tables of Food Composition in Japan.^19^

### Calculation of monetary diet cost

For both the DR and DHQ, monetary diet cost (Japanese yen/day) was calculated by multiplying the amount of each food reported (g/day) by the estimated price of the food (Japanese yen/g) and then summing the products (1 Japanese yen = 0.0047 pound sterling = 0.0059 euros = 0.0094 U.S. dollars in July 2008). The procedure for estimating costs was based on the assumption that all foods were purchased and then prepared and consumed at home.^12^,^14^ Calculations included correction for preparation and waste (e.g. trimming and peeling of vegetables and fruits, removal of bones and skin from fish).^6^,^14^ Costs of combined foods such as pizza were calculated using the prices of frozen equivalents.^14^,^15^ Water was excluded from calculation (two items in the DR and three items in the DHQ).^11^,^14^ The price of foods was obtained from two sources. The first was the National Retail Price Survey 2004.^23^ This survey was conducted in 167 villages, towns and cities, and average prices were calculated as mean values of all survey areas, weighted for population size. The second source was information on price from the websites of nationally distributed supermarket (Seiyu) and fast-food restaurant (McDonalds and Mister Donut) chains. When more than one price was available from the websites, the mean value was used.

To determine the price of individual food items, each food in the DR and DHQ was directly matched to foods appearing in the National Retail Price Survey. This procedure was used to determine the price of 656 of the 1297 items used in the DR (51%) and 120 of the 147 items used in the DHQ (82%). A total of 605 of the remaining 641 items in the DR for which a price value was not available in the National Retail Price Survey but which had a comparable food in terms of price (according to information on the websites) appearing in the National Retail Price Survey (47%) were assigned a value according to the comparable food. This procedure was also used to determine the price of 13 of the remaining 27 items in the DHQ (9%). For the remaining 36 items in the DR (3%) and 14 in the DHQ (10%) which had no price value and no comparable food in the National Retail Price Survey, prices were taken from the websites.

As the treatment of alcoholic beverages and noncaloric beverages in the calculation of monetary diet cost varies among studies,^3^–^17^ we used the following four calculation strategies: 1) all foods and beverages included; 2) alcoholic beverages excluded; 3) noncaloric beverages excluded; and 4) both alcoholic and noncaloric beverages excluded. Mean contributions to energy intake of the foods for which a price value was directly determined from the National Retail Price Survey, the foods which were assigned the price of a comparable food in the National Retail Price Survey, and the foods for which a price value was taken from the websites were 87%–91%, 5%–11%, and 2%–5%, respectively, depending on sex, dietary assessment method, and calculation strategy. The corresponding values for monetary diet cost were 81%–95%, 2%–17%, and 2%–6%, respectively.

While the misreporting of dietary intake, particularly by overweight subjects, is a serious problem associated with self-report dietary assessment methods,^24^ body mass index-dependent misreporting seems to be canceled by energy-adjustment, at least for potassium, sodium, and protein estimated from the DHQ.^25^ Thus, energy-adjusted values of monetary diet cost (by the residual and density models)^26^ were used in the present study. Because the results based on the residual model were quite similar to those based on the density model, we only present the results based on the energy-adjusted value of monetary diet cost by the density model (i.e. monetary cost of dietary energy (Japanese yen/4184 kJ)). The monetary cost of dietary energy of each food item in the DHQ (as well as the categorization of food groups) has been published elsewhere,^14^ except for the following 13 food items: three kinds of ice cream (regular 425, premium 959, and unspecified varieties 658 Japanese yen/4184 kJ), six alcoholic beverages (beer 1465, sake 746, shochu 497, shochu mixed with water or a carbonated beverages 533, whiskey 588, and wine 1235 Japanese yen/4184 kJ), and four noncaloric beverages (green and oolong tea 11, black tea 17, coffee 18, and sugar-free soft drinks 24 Japanese yen/100 g of edible weight).

### Statistical analysis

All statistical analyses were performed for women and men separately using SAS statistical software version 8.2 (SAS Institute Inc., Cary, NC, U.S.A). Distributions of monetary cost of dietary energy were evaluated for deviations from normality; because the variable was not strongly skewed, untransformed values were used. Mean and SD values for monetary cost of dietary energy were calculated for both DRs and DHQs. To assess seasonal variation, intraclass correlations were calculated using DRs (DR1, DR2, DR3 and DR4) and DHQs (DHQ1, DHQ2, DHQ3 and DHQ4) conducted in each season over a 1-year period. Intraclass correlations were also calculated between DHQs completed in the same season about one year apart (DHQ1 and DHQ5) to assess reproducibility of the DHQ.

To assess the comparability of the DR and DHQ, Pearson correlations between the mean of DRs1-4 and mean of DHQs1-4 were calculated. Pearson correlations were also calculated between the mean of DRs1-4 and DHQ1 to examine whether the DHQ (assessing dietary habits during the preceding month) is able to capture monetary cost of dietary energy over a longer period (i.e. one year). We used DHQ1 for this purpose because the answers provided in the other DHQs (administered after gaining experience of the DRs), but not DHQ1 (administered before this experience), may have been influenced by the attention to diet required to complete the DRs. Since random within-individual error in the measurement of any of the variables being compared tends to reduce correlation coefficients toward zero,^27^ correlations with the corrections for the attenuating effects of such measurement error in the 4 × 4-day DRs were also computed, as described elsewhere.^18^ Additionally, we calculated the percentage of subjects who were classified in the same, adjacent, or opposite quintile of monetary cost of dietary energy in the two different assessment methods. Further, the agreement between the two methods was assessed by the method proposed by Bland and Altman,^28^ using a plot of the difference between the two methods against the average of the two methods.

## Results

As shown in Table 1 (for DRs) and Table 2 (for DHQs), monetary cost of dietary energy was calculated from both dietary assessment methods conducted in each season over one year (DR1, DR2, DR3 and DR4 and DHQ1, DHQ2, DHQ3 and DHQ4) for assessing seasonal variations. Mean differences were within 6% for DRs and 9% for DHQs, and intraclass correlations ranged from 0.52 to 0.63 for DRs and 0.54 to 0.66 for DHQs. To assess the reproducibility of DHQ, the intraclass correlations between DHQs completed one year apart (DHQ1 and DHQ5) was calculated (Table 2). The intraclass correlations ranged from 0.50 to 0.64, with mean differences of less than 1%.

Comparability of the DR and DHQ for estimating monetary cost of dietary energy was assessed by using the value derived from DRs1- 4 and that derived from DHQs1- 4 (Table 3). Mean differences between DRs1-4 and DHQs1- 4 were within 8%. The Pearson correlations between DRs1- 4 and DHQs1- 4 ranged from 0.60 to 0.71. The percentage of subjects categorized into the same or adjacent quintiles was more than 71%, while the percentage categorized into the opposite quintile was less than 3%. Comparison of the first DHQ (DHQ1) with DRs1- 4 was also conducted to examine whether the DHQ (assessing dietary habits during the preceding month) is able to capture monetary cost of dietary energy over a longer period (i.e. one year) (Table 3). Mean differences between DRs1- 4 and DHQ1 were within 10% and Pearson correlations ranged from 0.41 to 0.61, while the percentage of subjects categorized to the same or adjacent and opposite quintiles was more than 61% and less than 4%, respectively.

**Figure 1.**
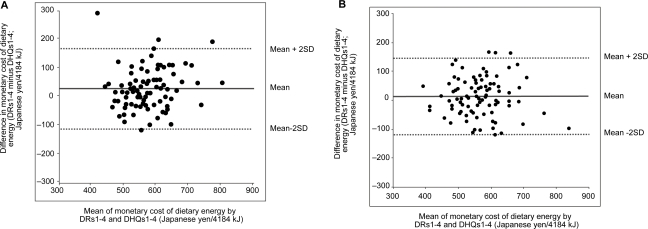
Bland-Altman plots assessing the agreement between 4-day weighed dietary records (DRs) and self-administered diet history questionnaires (DHQs) conducted in each season over one year (mean of DRs1-4 and mean of DHQs1-4, respectively) for monetary cost of dietary energy (calculated based on all foods and beverages) in 92 Japanese women (**a**) and 92 Japanese men (**b**).

Bland-Altman plots assessing the agreement between DRs1- 4 and DHQs1- 4 for monetary cost of dietary energy (calculated based on all foods and beverages) are shown in Figure 1. The mean difference (95% CI) between the two methods (DRs1- 4 minus DHQs1- 4) was 23.6 (9.2, 38.1) Japanese yen/4184 kJ for women and 12.8 (−0.9, 26.6) Japanese yen/4184 kJ for men, indicating relatively good agreement at the group level. The limits of agreement (mean difference ± 2SD of the difference) ranged from −115.6 to 162.9 Japanese yen/4184 kJ for women and −120.0 to 145.7 Japanese yen/4184 kJ for men, indicating somewhat moderate to poor agreement at the individual level. The plots indicated no tendency of consistent bias. Similar plots were observed when different cost calculation strategies or DHQ1 rather than DHQs1-4 were used (data not shown).

Important contributors to total monetary diet cost (based on DRs1-4 and DHQs1-4) were vegetables (12.0%–19.4%), fish and shellfish (17.0%–19.2%), meat (11.5%–12.8%), and noncaloric beverages (6.5%–10.0%), followed by confectioneries (4.3%–9.0%), fruits (5.1%–7.8%) and rice (5.8%–6.8%). In men, alcoholic beverages were also important contributors (12.6%–16.1%).

## Discussion

The present study of 92 Japanese women and 92 Japanese men showed reasonable comparability of monetary cost of dietary energy across DR and a DHQ for Japanese adults. Additionally, even a single administration of our DHQ (assessing dietary habits during the preceding month) appeared to relatively reasonably capture monetary cost of dietary energy over a longer period (i.e. one year), seemingly due to a relatively small seasonal variation in monetary cost of dietary energy as well as good reproducibility of DHQ. Because this is the first study to examine the comparability of monetary diet cost across dietary assessment methods, comparison of our results with others cannot be readily made. However, the comparability of DHQ and DR for estimating monetary cost of dietary energy observed here was similar to that for nutritional factors commonly studied in epidemiological studies with the use of dietary assessment questionnaires.^26^

The major contribution to total monetary diet cost in the present study came from perishable fresh foods such as vegetables, fish and shellfish, and meat. Consistent findings have been observed in several previous studies.^14^–^16^ This is reasonable given that transport, storage, and wastage costs are all high for perishable fresh produce. Although the question of whether alcoholic and noncaloric beverages should be included in the calculation of monetary diet cost has not been answered,^3^–^17^ the contribution of these beverages in the present study was not small. The treatment of these beverages in future research should thus be carefully considered, although the comparability of monetary cost of dietary energy here did not materially differ irrespective of the treatment of these beverages.

Several limitations of the present study should be mentioned. First, because of a lack of the true measure of monetary diet cost (i.e. actual food expenditure data), the present study unfortunately provides no information on the validity of monetary diet cost estimated based on food intake data derived from dietary assessment methods. Alternatively, the present study only provides information on the comparability of a DHQ and DR for estimating monetary diet cost. Thus, future investigation on the validity of monetary diet cost estimated from dietary intake data against true measure of monetary diet cost (e.g. a shopping diary and the collection of grocery till receipts supplemented by the recording of actual food consumption) is needed, although obtaining an accurate measure of food expenditure data at the individual level seems to be somewhat challenging.^12^

Second, both dietary assessment methods used in the present study (i.e. DR and DHQ) are not free from measurement error. However, it should be noted that errors in DR are thought to have lesser correlation with errors in DHQ, because the major sources of error associated with DHQ are limited food items, memory of food consumed, assessment of portion size, and interpretation of questions, while these sources of potential error are minimally shared with the DR method, which is open-ended, involves recording of foods as they are consumed, and involves direct weighing of food portions.^26^

Third, food prices were derived from the National Retail Price Survey and websites of nationally distributed supermarket and fast-food restaurant chains. Because this procedure provides a single cost value for a given food, without consideration of local, regional, or between-subject variations, it provides only an approximation of actual diet costs. Errors in the price values for foods will be shared by the DR and DHQ and may increase the observed correlations. Although this characteristic is common to standard nutrient databases, the actual diet cost may depend on where people live and shop, the number of people in the household (e.g. higher prices for the same food item for small households due to smaller packet size), or the extent to which people eat out at restaurants and takeaways.^16^ However, it should be noted that a similar methodology has been used in all previous studies^4^–^17^ with only one exception,^3^ as mentioned above.

Finally, the generalizability of these results may be limited, because the study evaluated one particular DHQ designed for use in Japan. Further, the sample size of this study was relatively small, and the subjects were not a representative sample of general Japanese but rather volunteers. Additional studies for other dietary assessment techniques in other populations would add valuable information on this topic.

To conclude, the present data indicate the reasonable comparability of monetary cost of dietary energy across DR and a DHQ for Japanese adults as well as the usefulness of a single administration of the DHQ for estimating monetary cost of dietary energy. The present findings may lend support to the practice of using dietary assessment questionnaires to estimate monetary diet cost.

## Figures and Tables

**Table 1.** Monetary cost of dietary energy (Japanese yen/4184 kJ) estimated from 4-day weighed dietary records (DRs) conducted in each season over one year (DR1, DR2, DR3 and DR4) and intraclass correlation (***r***) in 92 Japanese women and 92 Japanese men**^a^**.

	**DR1^b^**		**DR2^c^**		**DR3^d^**		**DR4^e^**		**Intraclass *r***

	**Mean**	**SD**	**Mean**	**SD**	**Mean**	**SD**	**Mean**	**SD**	
Women									
Including all foods and beverages	582	97	573	97	583	94	603	111	0.63
Excluding alcoholic beverages	572	92	564	93	574	91	591	107	0.60
Excluding noncaloric beverages	545	95	537	94	543	91	561	109	0.62
Excluding both alcoholic and noncaloric beverages	535	90	527	90	534	88	549	103	0.59
Men									
Including all foods and beverages	575	97	558	98	569	102	594	98	0.59
Excluding alcoholic beverages	549	96	536	92	540	91	558	89	0.52
Excluding noncaloric beverages	544	97	528	96	535	104	559	100	0.61
Excluding both alcoholic and noncaloric beverages	515	96	503	88	504	90	519	88	0.53

a 1 Japanese yen = 0.0047 pound sterling = 0.0059 euros = 0.0094 U.S. dollars in July 2008.

b Conducted in November and December 2002 (autumn).

c Conducted in February 2003 (winter).

d Conducted in May 2003 (spring).

e Conducted in August and September 2003 (summer).

**Table 2.** Monetary cost of dietary energy (Japanese yen/4184 kJ) estimated from self-administered diet history questionnaires (DHQs) conducted in each season over one year (DHQ1, DHQ2, DHQ3 and DHQ4) and that conducted one year after completion of DHQ1 (DHQ5), and intraclass correlation (***r***) in 92 Japanese women and 92 Japanese men**^a^**.

	**DHQ1^b^**		**DHQ2^c^**		**DHQ3^d^**		**DHQ4^e^**		**DHQ5^f^**		**Intraclass *r***	

	**Mean**	**SD**	**Mean**	**SD**	**Mean**	**SD**	**Mean**	**SD**	**Mean**	**SD**	**DHQs1-4**	**DHQ1 and DHQ5**
Women												
Including all foods and beverages	562	92	540	84	558	80	586	88	558	86	0.66	0.64
Excluding alcoholic beverages	550	83	531	80	545	80	574	88	548	84	0.63	0.61
Excluding noncaloric beverages	505	87	489	84	503	81	535	88	504	85	0.66	0.64
Excluding both alcoholic and noncaloric beverages	492	80	479	80	489	80	522	84	493	84	0.64	0.62
Men												
Including all foods and beverages	555	99	548	99	544	97	591	92	556	95	0.59	0.51
Excluding alcoholic beverages	512	82	506	90	504	80	546	74	513	80	0.54	0.50
Excluding noncaloric beverages	511	99	507	95	499	100	548	92	515	97	0.64	0.57
Excluding both alcoholic and noncaloric beverages	463	81	459	84	454	80	498	76	468	80	0.61	0.57

a 1 Japanese yen = 0.0047 pound sterling = 0.0059 euros = 0.0094 U.S. dollars in July 2008. DHQ is designed to assess dietary habits during the preceding month.

b Conducted in November 2002 (autumn).

c Conducted in February 2003 (winter).

d Conducted in May 2003 (spring).

e Conducted in August and September 2003 (summer).

f Conducted in November 2003 (autumn).

**Table 3.** Monetary cost of dietary energy (Japanese yen/4184 kJ) estimated from 4-day weighed dietary records (DRs) and self-administered diet history questionnaires (DHQs) conducted in each season over one year (mean of DRs1-4 and mean of DHQs1-4, respectively) and the Pearson correlation (***r***) and percentage of subjects classified in the same, adjacent, and opposite quintiles between the mean of DRs1-4 and that of DHQs1-4 and between mean of DRs1-4 and the first DHQ (DHQ completed before DRs; DHQ1) in 92 Japanese women and 92 Japanese men**^a^**.

	**Mean of DRs1-4^b^**		**Mean of DHQs1-4^c^**		**Mean of DRs1-4 and mean of DHQs1-4**					**Mean of DRs1-4 and DHQ1**				

					**Pearson *r***		**Cross-classification (%)**			**Pearson *r***		**Cross-classification (%)**		

	**Mean**	**SD**	**Mean**	**SD**	**Crude**	**Corrected^d^**	**Same quintile**	**Adjacent quintile**	**Opposite quintile**	**Crude**	**Corrected^d^**	**Same quintile**	**Adjacent quintile**	**Opposite quintile**
Women														
Including all foods and beverages	585	85	561	76	0.63	0.64	38	38	0	0.59	0.60	36	37	1
Excluding alcoholic beverages	575	81	550	71	0.59	0.61	42	34	0	0.55	0.56	41	33	1
Excluding noncaloric beverages	547	83	508	75	0.66	0.67	42	34	0	0.60	0.61	39	32	1
Excluding both alcoholic and noncaloric beverages	536	77	496	71	0.62	0.63	42	29	0	0.55	0.57	37	37	1
Men														
Including all foods and beverages	573	82	561	83	0.68	0.69	42	36	0	0.51	0.52	29	42	1
Excluding alcoholic beverages	545	74	517	68	0.58	0.60	33	41	1	0.40	0.41	27	35	3
Excluding noncaloric beverages	541	83	517	85	0.70	0.71	37	42	0	0.56	0.57	32	43	1
Excluding both alcoholic and noncaloric beverages	509	73	469	70	0.58	0.60	28	49	2	0.45	0.46	27	39	3

a 1 Japanese yen = 0.0047 pound sterling = 0.0059 euros = 0.0094 U.S. dollars in July 2008. DHQ is designed to assess dietary habits during the preceding month.

b Conducted in November and December 2002 (autumn), February 2003 (winter), May 2003 (spring) and August and September 2003 (summer).

c Conducted in November 2002 (autumn), February 2003 (winter), May 2003 (spring) and August and September 2003 (summer).

d Corrected for seasonal variation in DRs.
